# Three novel quantum-inspired swarm optimization algorithms using different bounded potential fields

**DOI:** 10.1038/s41598-021-90847-7

**Published:** 2021-06-02

**Authors:** Manuel S. Alvarez-Alvarado, Francisco E. Alban-Chacón, Erick A. Lamilla-Rubio, Carlos D. Rodríguez-Gallegos, Washington Velásquez

**Affiliations:** 1 Faculty of Electrical and Computer Engineering, Escuela Superior Politécnica del Litoral, EC090112 Guayaquil, Ecuador; 2 Faculty of Natural Science and Mathematics, Escuela Superior Politécnica del Litoral, EC090112 Guayaquil, Ecuador; 3 Solar Energy Research Institute of Singapore (SERIS), National University of Singapore (NUS), Singapore, 117574 Singapore

**Keywords:** Engineering, Mathematics and computing

## Abstract

Based on the behavior of the quantum particles, it is possible to formulate mathematical expressions to develop metaheuristic search optimization algorithms. This paper presents three novel quantum-inspired algorithms, which scenario is a particle swarm that is excited by a Lorentz, Rosen–Morse, and Coulomb-like square root potential fields, respectively. To show the computational efficacy of the proposed optimization techniques, the paper presents a comparative study with the classical particle swarm optimization (PSO), genetic algorithm (GA), and firefly algorithm (FFA). The algorithms are used to solve 24 benchmark functions that are categorized by unimodal, multimodal, and fixed-dimension multimodal. As a finding, the algorithm inspired in the Lorentz potential field presents the most balanced computational performance in terms of exploitation (accuracy and precision), exploration (convergence speed and acceleration), and simulation time compared to the algorithms previously mentioned. A deeper analysis reveals that a strong potential field inside a well with weak asymptotic behavior leads to better exploitation and exploration attributes for unimodal, multimodal, and fixed-multimodal functions.

## Introduction

Gradient-based and Hessian-based algorithms are widely use in the literature to solve optimization problems in engineering. This is due to their computational efficiency, which typically require little problem-specific parameter tuning^1^. The Gradient-based and Hessian-based algorithms employ iterative process that involves multivariate scalar function that packages all the information of the partial derivatives of the objective function (i.e., Gradient function or Hessian matrix) to reach the solution. Some of the most common methods in this category are: interior point, quasi-Newton, descent gradient, and conjugate gradient^1^. The main drawback with such algorithms includes volatility to reach local optima solutions, difficulty in solving discrete optimization problems, intricacy in the implementation for some complex optimization multivariable problems, and susceptibility to numerical noise^2^. To tackle the presented deficiencies, some authors suggest the use of metaheuristics optimization techniques.

**Figure 1:**
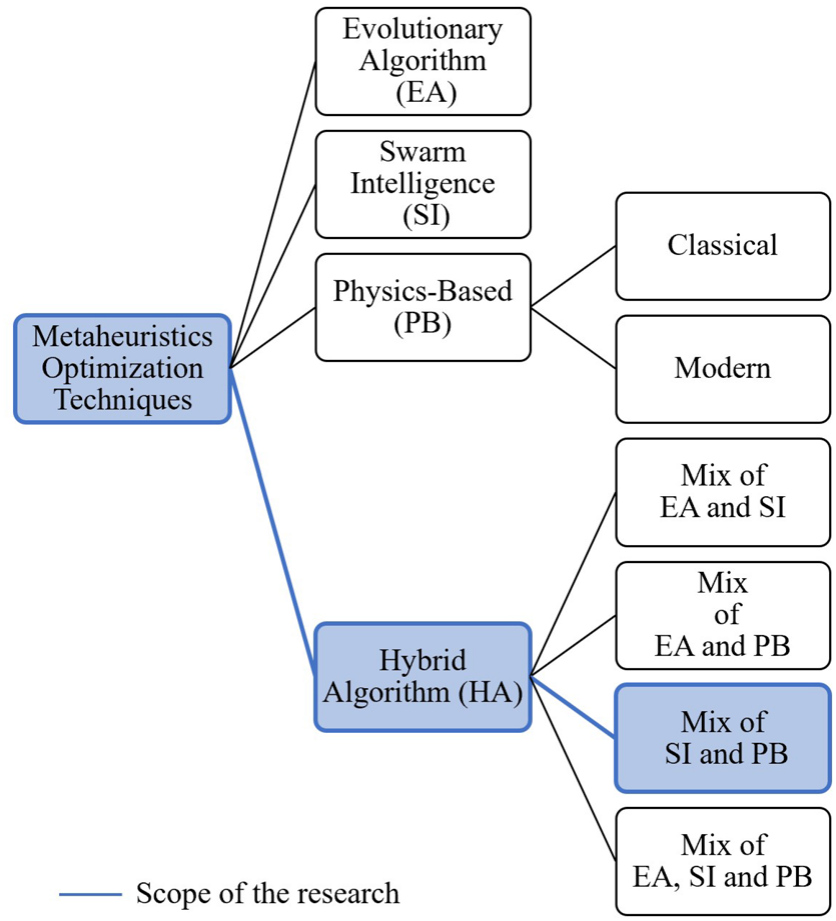
Classification of metaheuristics optimization techniques.

In the last two decades, the use of metaheuristics optimization techniques to solve complex, multimodal, high dimensional and nonlinear engineering problems have become very popular. This is attributed to their simplicity of implementation, straightforward adaptability to solve optimization problems, and robust search capability to achieve effective global optima^3^. Even though there are different metaheuristics optimization techniques, these can be classified into four main groups as presented in Fig. 1. The first group is called evolutionary algorithm (EA). The theory behind the EA is based on the evolution in nature. In this field, Genetic Algorithm (GA) emerge as the most popular. GA was proposed by Holland in 1992^4^. GA is inspired by the Darwin evolution theory^4^, in which a population (solution candidates) evolves (best solution) by crossover and mutation processes. In this way, the solution to the global optima in every iteration (next generation) is assured. Recently, an extensive recompilation of GA applications can be found in^5^. Other advanced EAs are Biogeography-Based Optimizer^6^, Clonal Flower Pollination^7^, Fuzzy Harmony Search^8^, Mutated Differential Evolution^9^, Imperialist Competitive (2017)^10^, and Deep Learning with Gradient-based optimization^11^.

The second metaheuristic group corresponds to swarm intelligence (SI). These algorithms incorporate mathematical models that describe the motion of a group of creatures in nature (swarms, schools, flocks, and herds) based on their collective and social behavior. The most well-known SI algorithm presented in the literature is the Particle Swarm Optimization (PSO), and was proposed by Kennedy and Eberhart in 1995^12^. In general, SI algorithms initialize with multiple particles located in random positions (solution candidates). The particles look to enhance their position based their position based on their own best positions obtained so far and best particle of the swarm. The motion process is repeated (iterations) until most of the particle converge to the same position (best solution). The theory behind SI have been exploited, resulting in novel innovative optimization techniques (i.e. Dynamic Ant Colony Optimization (2017)^13^, Bacterial Foraging (2016)^14^, Fish School Search (2017)^15^, Moth Firefly (2016)^16^, and Chaotic Grey Wolf^17^) for different engineering applications.

The third metaheuristic group is physics-based (PB). Their formulation involves any physical concept used to describe the behavior through space and time of the matter. PB algorithms can be classified into two main classes: classical and modern. The term ‘classical’ refers to the optimization techniques that employs classical physics in their formulations to reach the optima global. In this branch, fits the Greedy Electromagnetism like^18^, Improved Central Force^19^, Multimodal Gravitational Search^20^, Exponential Big Bang-Big Crunch (BB-BC)^21^, and Improved Magnetic Charged System Search^22^ algorithms. On the other hand, the term ‘modern’ refers to the algorithms that employs quantum physics to determine the optima global. Some of the recent algorithms that fit in this branch are Neural Network Quantum States^23^, Adiabatic Quantum^24^, and Quantum Annealing^25^ optimizations. PB algorithms’ optimization process starts with a random initialization of the matter’s position (solution candidates). Then, depending on the physical interaction (i.e. kinematic, dynamic, thermodynamic, hydrodynamic, momentum, energy, electromagnetism, quantum mechanics, etc.) defined in the search space, the particles improve their position (best solution) and the process is repeated until certain physical rules are satisfied.

The last metaheuristic group is Hybrid algorithm (HA). This group combines the characteristics of the previous metaheuristics groups to bring new optimization techniques. These algorithms can be classified into four main groups: EA-SI (i.e. Evolutionary Firefly^26^), EA-PB (i.e. Harmony Simulated Annealing Search^27^), SI-PB (i.e. Big Bang-Big Crunch Swarm Optimization^28^) and EA-SI-PB (i.e. Electromagnetism-like Mechanism with Collective Animal behavior search^29^). Particularly, the research scope lies on the SI-PB group since it combines the quantum physics concepts and swarm particle behavior.

The recent literature presents new approaches that use the concepts of quantum mechanics and swarm intelligence for different applications. For instance^30^, exhibits a Quantum-inspired Glow-worm Swarm Optimisation (QGSO) to minimize the array style with maximum relative sidelobe level of array (discrete optimization problem). The algorithm employs the concepts of quantum bits combined with the mathematical behaviour of social glow-worm swarm to determine the best solution in terms of the position of the best quantum glow-worm. Authors in^31^, propose a novel Accelerated Quantum Particle Swarm Optimization (AQPSO) that uses the concept of quantum mechanics to derive an expression that deals with the position of the quantum particle trapped in a delta potential well. In order to accelerate the convergence process, the inclusion of an odd number of observers greater than unity is incorporated to the model. The AQPSO shows high performance resulting in the application of different power systems application such as optimal placement of static var compensators for maximum system reliability^31^, maximization of savings due to electrical power losses reduction^32^, and optimal maintenance schedule to minimize the operational risk of static synchronous generators^33^ and power generators^34^. In^35–37^, quantum ant colony algorithm is used to solve path optimization problems. In this algorithm, every ant carries a group of quantum bits to represents the position of its own. The ants move according through quantum rotation gates, which lead to an improvement in their position. As presented, there is plenty of evidence that demonstrate the computation effective robustness of the quantum SI-PM algorithms. Most of them are driven by quantum bits^30,35–37^, and quantum potential wells^31,38,39^. Nevertheless, to the best of our knowledge, there is no quantum SI-PM in the literature mimicking a quantum particle swarm bounded by Lorentz, Rosen–Morse, and Coulomb-like Square Root potential fields. This fact encourages the attempt to propose three novel algorithms and investigate its abilities in solving benchmark optimization problems.

**Figure 2:**
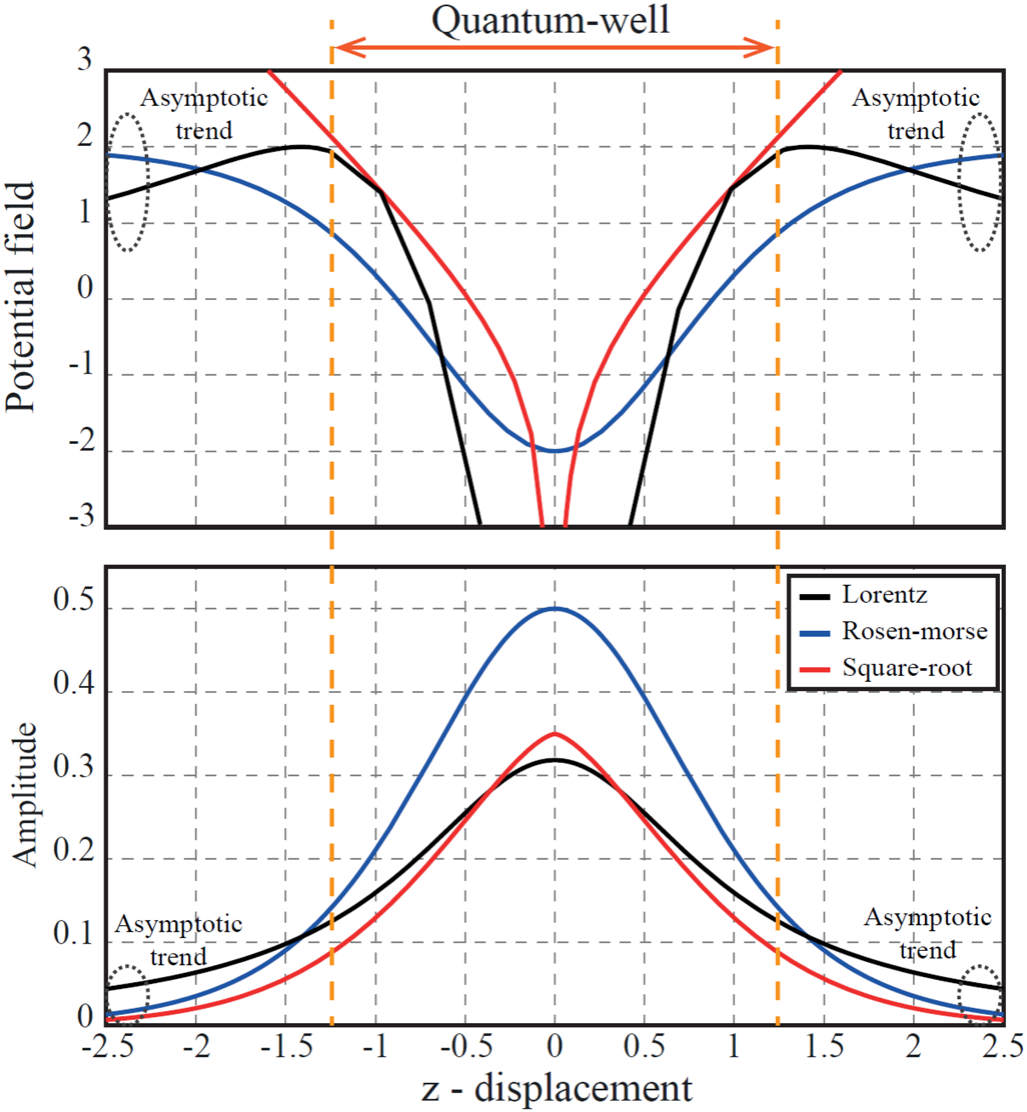
Probability density functions associated with the potential fields.

The motivation of this research lies on the No Free Lunch (NFL) theorem, which states that “any two algorithms are equivalent when their performance is averaged across all possible problems”^40^. The given statement infers that there is not a best algorithm able to solve any optimization problem. Some algorithms may show effective performance for a set of problems; however, the same algorithms can result inefficient for a different set of problems. Therefore, NFL open a pathway to improvement of the existing approaches. Given the foregoing, this paper proposes three novel hybrid metaheuristics optimization techniques inspired by the movement behaviour of quantum particle swarm bounded in three different potential fields: Lorentz, Rosen-Morse, and Coulomb-like Square Root. The given potentials fields are considered due to their simplicity in the analytical solution to the Schrödinger equation, which are widely studied in the Physics literature^41–43^. Moreover, these potentials fields offer certain features that allow to predict the qualitative behaviour of the proposed algorithms in terms of exploitation and exploration. The base for this statement lies in the probability density function (solution to the Schrödinger equation), which presents two regime behaviour. The first regime is in between the limits of the quantum well, while second regime is related to the asymptotic trend at $(z \rightarrow \pm \infty )$, as presented in Fig. 2. The local amplitude of the probability density function (local “height” or probability) represents the strength of the potential in a region of space. In this sense, the behaviour of the probability density function in between the limits of the quantum well illustrates the probability of the particle to be near the local attractor that is associated with the exploration capabilities of the search algorithm^44^. It is important to highlight that the exploration is defined as the ability of examining a promising area(s) as broadly as possible. By defining a “promising area” as the region near the local attractor, then more points are expected to be searched in the promising area if more probability weight is given locally. This means that a higher amplitude (near zero) of the probability density function leads to a more thoroughly/broadly search in that region, giving rise to more exploration. Hence, the probability density function that is expected to produce the most exploration in the search algorithm is the Rosen Morse probability density function, followed by the Lorentz and the Coulomb like Square Root probability density functions. The behaviour of the probability density function at $(z\rightarrow \pm \infty )$ is associated with the global search capabilities of the algorithm, known in this manuscript as exploitation. Therefore, a slowly decaying probability density function (weak potential at $(z\rightarrow \pm \infty )$) will be expected to exhibit high exploitation, i.e. further values from the local attractor will be searched with non-negligible probability^44^. In this sense it is expected the Lorentz probability density function to present the best exploitation, followed by the Rosen Morse and the Coulomb-like Square Root probability density function.

To verify the computational robustness of the proposed approach, several benchmark functions are solved. Then, the results are compared with the ones obtained by particle swarm optimization, genetic algorithm, and firefly algorithms. The rest of the paper is organized as follows: “Quantum Particle Swarm Optimization general formulation” section presents quantum concepts that describe the scenario of the particle swarm. “Time independent Schrödinger equation and particle position” section describes the nature of a quantum particle in a bounded potential field. “Quantum-inspired optimization algorithms” section exhibits the quantum-inspired proposed optimization algorithms. “Case study” section describe the case study used to test the efficacy of the proposed algorithms. In “Results” section, the results are analysed and discussed. Finally, “Discussion and conclusion” section incorporates the conclusions.

## Methodology

### Quantum particle swarm optimization general formulation

**Figure 3:**
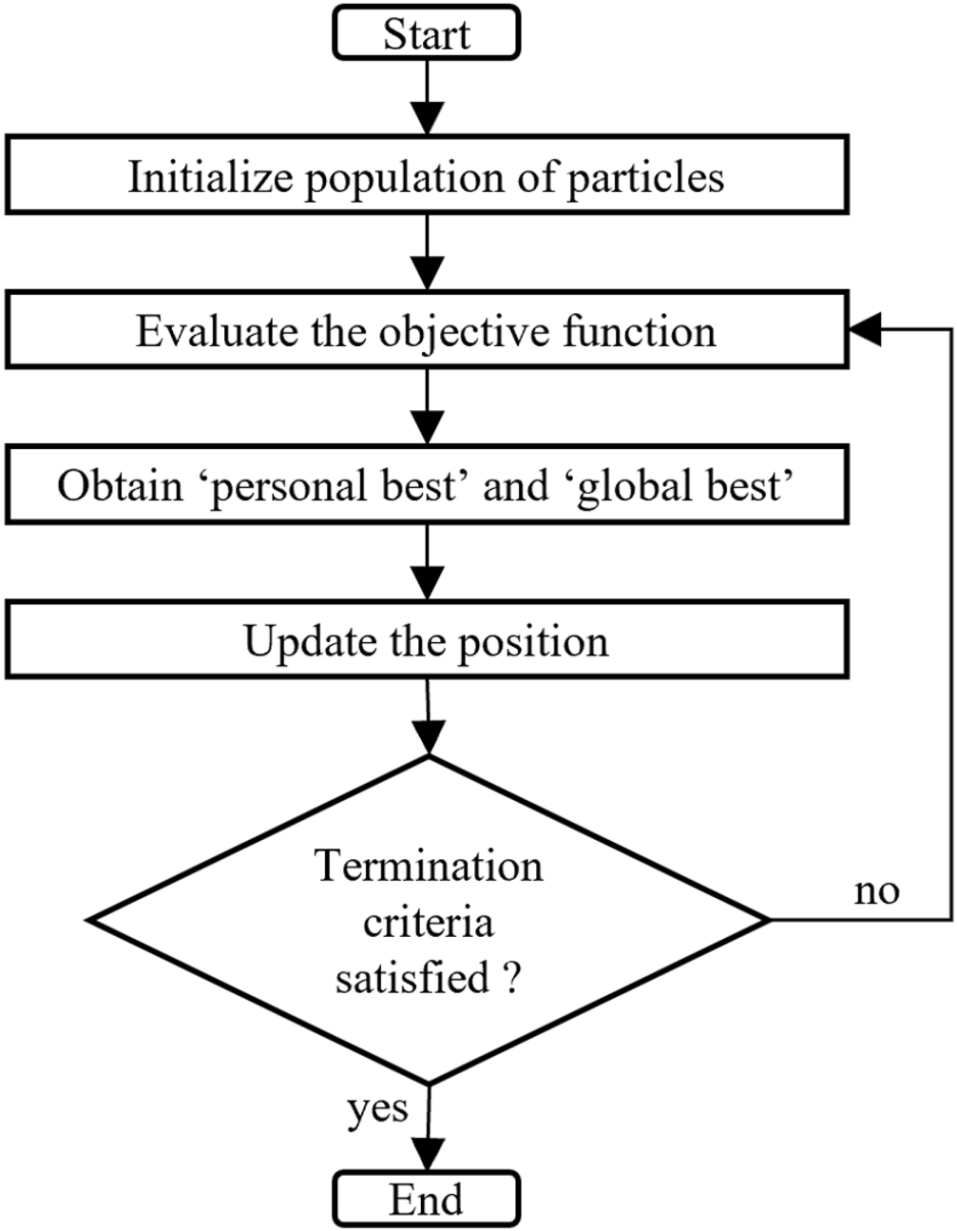
QPSO flowchart.

Quantum particle swarm optimization (QPSO) is an advanced heuristic optimization technique that employs the concept of quantum particle motion to reach the optimal solution. QPSO follows the process described in Fig. 3. The process starts defining the initial population of the particles SS and total number of iterations *MaxIt*. The position of the particle (*x*) represents a solution candidate to the optimization problem; thus, it can be used to evaluate the objective function.

**Figure 4:**
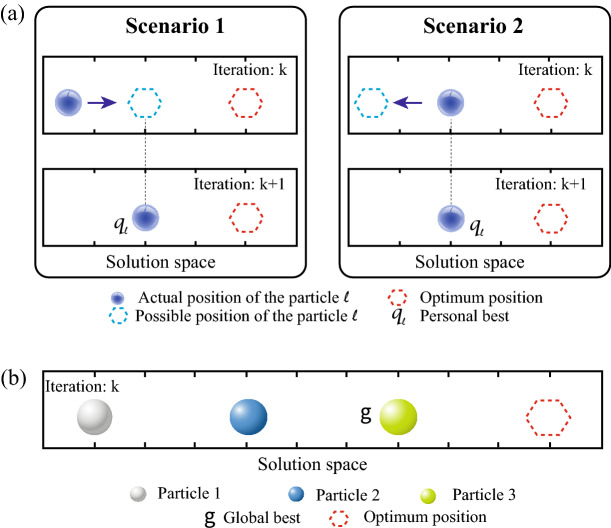
(**a**) Memory attribute of the particle. (**b**) Communication attribute of the particle.

The next step is to identify the positions called ‘personal best’ and ‘global best’. In this step is relevant to consider two specific attributes of the particle, which are related to memory and communication. The memory attribute refers to the ability to save the best position of the particle by comparing its actual position with the position after the motion. For instance, Fig. 4a shows two scenarios of particle motion. In scenario 1, the particle has the possibility to move close to the optimum position, therefore, it proceeds to move and saves this position as its best position. In scenario 2, the particle has the possibility to move far from the optimum position, therefore, it will not move and saves its actual position as its best position. The memory attribute is known as ‘personal best’ and denoted by *q*^45,46^. The communication attribute refers to the ability to save the particle with the best position among the swarm. Figure 4b shows a swarm with three particles, resulting in ‘particle 3’ as the best particle since is the one nearest to the optimum position. The communication attribute is known as ‘global best’ and is denoted by *g*^45,46^.

The process continues with the update of the particles position. For the given purpose, the general mathematical topology of the swarm intelligence optimization is employed. This is as follows^47^:

1 $$\begin{aligned} x_{\ell }^{(k+1)}=z\left( x_{\ell }^{(k)},V\right) +f_1 \left( w_1,u_1 \right) \left( q_{\ell }^{(k)}-x_{\ell }^{(k)} \right) +f_2 \left( w_2,u_2 \right) \left( g^{(k)}-x_{\ell }^{(k)} \right) \end{aligned},$$

where *z* represent the displacement of a particle and is a function that depends on $x_{\ell }^{(k)}$ and *V*, which represent the actual position *x* of the particle $\ell$ at iteration *k* and the physics phenomena that drives the movement of the particle, respectively. The functions $f_1$ and $f_2$ correspond to the nature of the swarm intelligence, in which $f_1$ drives the new position of the particle towards the local optima particle position q, while $f_2$ associates the new position of the particle with the global optima particle position *g*. To avoid traps (‘local optima’) that may appear in the objective function, the authors of the first swarm intelligence^12^ introduced random numbers *u* and coefficients of acceleration *w* such that $0 \le w \le 2$. The suffix ‘1’ and ‘2’ is used to refer to local and global position, respectively.

A simplification of the general formulation of the swarm intelligence optimization, is proposed by authors in^47^. The model is based on a trajectory analysis in which the best position of the particle is localized in the search space that lies in between the best local and global position. Authors in^31^ refer to this term as local attractor $D_{\ell }^{(k)}$, and is used to guide the particle towards a better position. Such model has the form^47^:

2 $$\begin{aligned} x_{\ell }^{(k+1)}= \,& {} z(x_{\ell }^{(k)},V)+D_{\ell }^{(k)}, \nonumber \\ D_{\ell }^{(k)}= &\, {} \left( (r_1 u_1)/(r_1 u_1+r_2 u_2 )\right) q_{\ell }^{(k)}+(1-(r_1 u_1)/(r_1 u_1+r_2 u_2 )) g^{(k)}. \end{aligned}$$

Reference^48^ presents that for the scenario of a quantum particle trapped in a bounded field, the phenomena that drives the movement of the particle is given by the relative width a and a function that depends on the potential well being used, such that^48^

3 $$\begin{aligned}&z\left( x_{\ell }^{(k)},V\right) =af(V), \nonumber \\&a=\left| x_{\ell }^{(k)} -\frac{1}{SS} \sum _{\ell =1}^{SS} q_{\ell }^{(k)} \right| \end{aligned},$$

where *SS* is the total number of particles. This last formulation is employed to estimate the new position of the particle. The function *f*(*V*) is analyzed in the following sections of the manuscript.

The last step is to verify the termination criterion using the total number of iterations *MaxIt*, and convergence tolerance value $\xi$. The process finishes if one of the conditions given in Eq. (4) is satisfied^49^.

4 $$\begin{aligned} Convergence~criteria:\left\{ \begin{matrix} k = \textit{MaxIt}\\ \left| \sum _{\ell =1}^{SS} q_{\ell }^{(k)} - SS g^{(k)} \right| \le \xi \end{matrix}\right. \end{aligned}.$$

### Time independent Schrödinger equation and particle position

QPSO algorithm can be described through the quantum behaviour of motion of particles. In particular, the case of a particle moving in a bounded potential field is considered of great interest. Under the given context, particles’ states are depicted by the probability density function $|\psi ({\vec {r}},t)|^2$, in the search space ${\vec {r}}$, at time *t*. The probability density function must satisfy^49^.

5 $$\begin{aligned} \int _{-\infty }^{+\infty } \left| \psi ({\vec {r}},t) \right| ^2 d{\vec {r}} = 1 \end{aligned}.$$

In order to determine the position of the particle, a measurement is required. For this purpose, a random number generation simulation is performed $u=rand(0,1)$. In this sense, the wave function squared must be normalized with respect to its maximum value, such that

6 $$\begin{aligned} \frac{\left| \psi ({\vec {r}},t) \right| ^2}{max \left( \left| \psi ({\vec {r}},t) \right| ^2\right) } = u \end{aligned}.$$

On the other hand, the time evolution of the wave function $\psi ({\vec {r}},t)$ of a quantum system is generally described by the Schrödinger equation^49–51^:

7 $$\begin{aligned} {\widehat{H}}\psi ({\vec {r}},t)=i\hbar \frac{\partial }{\partial t}\psi ({\vec {r}},t) \end{aligned}.$$

The formulation presented in Eq. (7) is also called the time-dependent Schrödinger equation. In this equation, ${\widehat{H}}$ is the Hamiltonian operator and $\hbar$ is the reduced Planck Constant. Nevertheless, for the purpose of this research, a slowly varying process (adiabatic) in which the eigen estate at $E=0$ of the particle change accordingly with the evolution of the potential *V* is considered. Then, the one-dimension stationary Schrödinger equation can be used and written as follows^50,51^:

8 $$\begin{aligned} \frac{\hbar ^2}{2m}\frac{\partial ^2 }{\partial z^2}\psi (z) - V(z)\psi (z) = 0 \end{aligned}.$$

The last formulation is relevant for the derivation of the term *f*(*V*) required in Eq. (3).

### Quantum-inspired optimization algorithms

The following analysis considers wave functions that belongs to the Hilbert space ($\psi (z) \in {\mathcal {H}}$), that is, the wave function squared $\left| \psi (z) \right| ^2$ must be normalized with the boundary condition stablished in Eq. (9)^50,51^.

9 $$\begin{aligned} \lim _{z\rightarrow \pm \infty }\psi (z)=0 \end{aligned}.$$

**Figure 5:**
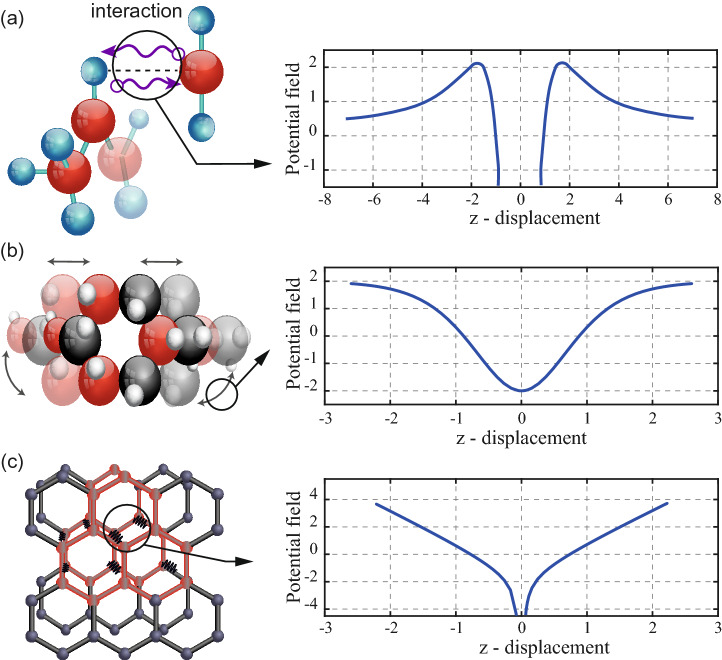
(**a**) Lorentz potential field to model donor acceptor interaction. (**b**) Rosen–Morse potential field to model molecular vibrations. (**c**) Coulomb-like square root potential field to model the electron confinement in graphene.

The proposed quantum-inspired optimization algorithms follow the methodology described in Fig. 1. In the methodology it is required to determine the term *f*(*V*), and complete the formulation presented in Eq. (3). In the following, *f*(*V*) is derived for each proposed potential field, which are shown in Fig. 5.

#### Lorentz potential field (QPSO-LR)

The following algorithm is inspired in the localization of electrons in bonds formation. This occurs when there is an interaction between electron–donor and electron–acceptor atoms, as presented in Fig. 5a. The potential field that describes this phenomenon is mathematically described as given in Eq. (10)^52^.

10 $$\begin{aligned} V(z)=\frac{\hbar ^2(2z^2 - a^2)}{2m(z^2 + a^2)^2} \end{aligned}.$$

The displacement of the particle under the Lorentz potential field is obtained by replacing Eq. (10) in Eq. (8), resulting in Eq. (11).

11 $$\begin{aligned} \frac{\hbar ^2}{2m}\frac{\text {d}^2 \psi (z)}{\text {d} z^2}- \frac{\hbar ^2(2z^2 - a^2)}{2m(z^2 + a^2)^2\psi (z)}=0 \end{aligned}.$$

The solution for the Second Order Linear Ordinary Differential Equation (SLODE) presented in Eq. (11) has the form given in Eq. (12).

12 $$\begin{aligned} \psi (z)=C_1\frac{1}{\sqrt{a^2+z^2}}+C_2\frac{3a^2z+z^3}{3\sqrt{a^2+z^2}} \end{aligned}.$$

By applying the boundary condition given in Eq. (9), the result is:

13 $$\begin{aligned} C_1=\sqrt{\frac{a}{\pi }}; C_2=0 \end{aligned}.$$

Then,

14 $$\begin{aligned} \psi (z) = \sqrt{\frac{a}{\pi (a^2+z^2)}} \end{aligned}.$$

By replacing Eq. (14) in Eq. (6) and solving for *z*, the result is as follows:

15 $$\begin{aligned} z=a\sqrt{\frac{1-u}{u}} \end{aligned}.$$

By replacing Eq. (15) in Eq. (3), the conclusion is:

16 $$\begin{aligned} f(V)=\sqrt{\frac{1-u}{u}} \end{aligned}.$$

#### Rosen–Morse potential field (QPSO-RM)

The Rosen-Morse potential field can be used to model the vibration energy spectra produced by the interaction of atoms in a diatomic molecule^53,54^, as presented in Fig. 5b. A generalized form of the Rosen-Morse potential field is given by Eq. (17).

17 $$\begin{aligned} V(z)=\frac{\hbar ^2\left[ \tan h^2(z/a) - \sec h^2(z/a) \right] }{a^22m} \end{aligned}.$$

The displacement of the particle under the Rosen–Morse potential is obtained by replacing Eq. (17) in Eq. (8), resulting in Eq. (18).

18 $$\begin{aligned} \frac{\hbar ^2}{2m}\frac{\text {d}^2 \psi (z)}{\text {d} z^2}- \frac{\hbar ^2}{2m}\frac{\left[ \tan h^2(z/a) - \sec h^2(z/a) \right] }{a^2} \psi (Z)=0 \end{aligned}.$$

Using Eq. (9) to normalize the solution to Eq. (18) and solving the SLODE, the result is given by (19).

19 $$\begin{aligned} \psi (z)=\frac{1}{\sqrt{2a}}\sec h(z/a) \end{aligned}.$$

Plugging in Eq. (19) back into Eq. (6) and solving for *z*, the result is as follows:

20 $$\begin{aligned} z = a \sec h^{-1}(\sqrt{u}) \end{aligned}.$$

Substituting Eq. (20) in Eq. (3), the conclusion is

21 $$\begin{aligned} f(V) = \sec h^{-1}(\sqrt{u}) \end{aligned}.$$

#### Coulomb-like square root field (QPSO-CS)

A potential that contains an inverse square root and a linear symmetric potential is evaluated. The Coulomb-like square root potential field is commonly employed to model the electron confinement in graphene^55^, as presented in Fig. 5c. Mathematically, the Coulomb-like square root potential field can be written as given in Eq. (22).

22 $$\begin{aligned} V(z)=\frac{\hbar ^2}{2m}\left[ -\frac{0.4}{a^{3/2}}\left| z \right| ^{-0.5} + \frac{0.6}{a^3}\left| z \right| \right] \end{aligned}.$$

Similarly, the displacement of the particle under the Coulomb-like Square Root potential is obtained by replacing Eq. (22) in Eq. (8), which leads to Eq. (23):

23 $$\begin{aligned} \frac{\hbar ^2}{2m}\frac{\text {d}^2 \psi (z)}{\text {d} z^2}- \frac{\hbar ^2}{2m}\left[ -\frac{0.4}{a^{3/2}}\left| z \right| ^{-0.5} + \frac{0.6}{a^3}\left| z \right| \right] \psi (Z)=0 \end{aligned}.$$

Using Eq. (9) to normalize the solution to Eq. (23) and solving the SLODE, the result is given by Eq. (24).

24 $$\begin{aligned} \psi (z)=\frac{1}{1.69\sqrt{a}}e^{-\left( \frac{\left| z\right| }{a} \right) ^{3/2}} \end{aligned}.$$

Plugging in Eq. (24) back into Eq. (6) and solving for *z*, the result is as follows:

25 $$\begin{aligned} z = a\left[ ln\left( \frac{1}{u} \right) \right] ^{2/3} \end{aligned}.$$

Substituting Eq. (25) in Eq. (3), the conclusion is:

26 $$\begin{aligned} f(V) = \left[ ln\left( \frac{1}{u} \right) \right] ^{2/3} \end{aligned}.$$

The implementation of the proposed optimizations techniques is presented in Algorithm 1. Notice that the main difference among the proposed algorithms lies in step 11, in which the particle updates its position. This is because the particle follows a trajectory that mainly depends on the boundary potential field defined in *f*(*V*). 
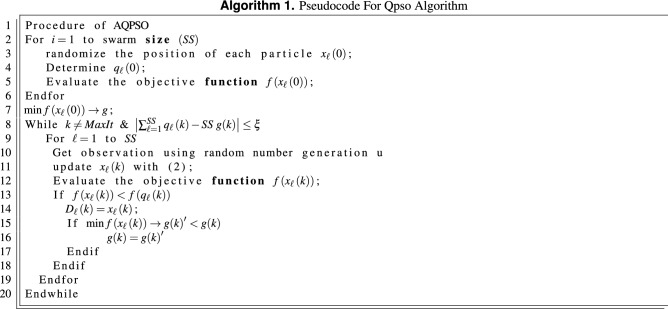


### Case study

To show the performance of the proposed algorithms (QPSO-LR, QPSO-RM and QPSO-LR), 24 benchmark functions are used^56^. The benchmark functions are categorized by unimodal, multimodal and fixed-dimension multimodal that are mathematically described in Table 7, 8 and 9, respectively. Unimodal functions are used to analyze the impact of the algorithms when there is one minimum value in a certain interval. In contrast, multimodal functions are utilized to analyze the algorithms in the presence of several local minima through the search space. The simulation incorporates for the unimodal and multimodal functions a dimension (total of variables) of 30, while for fixed-dimension multimodal is as shown in Table 9. Concerning the population size and total number of iterations, these are 50 and 1000, respectively. To corroborate the significance of the results, a total of 30 experiments (simulations) are conducted. All the algorithms are tested in MATLAB R2020a and numerical experiment is set up on Intel Core (TM) i7-6500 Processor, 2.50GHz, 8 GB RAM.

## Results

The performance of QPSO-LR, QPSO-RM and QPSO-LR is measured in terms of exploitation (accuracy and precision), exploration (search speed and acceleration) and simulation time. In addition, to explore the advantages of the proposed algorithms, the same optimization problems are solved using particle swarm optimization (PSO), genetic algorithm (GA), and firefly optimization (FFO). The results are shown as follows.

### Exploitation: accuracy and precision

The exploitation refers to the local search capability around the promising regions. This can be quantified based on two statistics metrics: accuracy $(\delta )$ and precision $(\phi )$. The term accuracy is defined as the absolute value of the difference between the average value and the true value (reference value) of the quantity being measured, that is, the closeness of the measurements to the true value. On the other hand, the term precision indicates the closeness of the measurements to each other. The introduced terms can be mathematically obtained using the true value $(x_{opt})$, $({\bar{x}})$), and standard deviation $(\sigma )$ of a set of data, as given in Eq. (27) and Eq. (28), respectively^57^.

27 $$\begin{aligned}&\delta =\left| x_{opt}-{\bar{x}}\right| \end{aligned},$$

28 $$\begin{aligned}&\phi =\left| \sigma /{\bar{x}}\right| \end{aligned}.$$

Using the data obtained from the experiments performed with each optimization technique, the mean and standard deviation can be obtained. Then, by the employment of (27) and (28), the accuracy and the precision of each optimization technique are obtained. Tables 1, 2 and 3 shows the statistical metrics for unimodal, multimodal, fixed-multimodal benchmark function, accordingly (best values are highlighted in blue). The results reveal that for each algorithm there is a better match in terms of accuracy and precision depending on the function. Focusing on the unimodal benchmark functions $(f_1-\ f_8)$, QPSO-LR presents the best accuracy for $f_1,\ f_2,\ f_4,\ f_6,\ f_7,$ followed by GA in functions $f_1,\ f_2,\ f_6,\ f_8$ and PSO for the functions $f_4$ and $f_7$. Likewise, if only precision is considered, there is considerable variation in the algorithms with respect to the functions, e.g., GA is the most precise for $f_1,\ f_2,\ f_7$, while for $f_4$ and $f_6$ the QPSO-RM and QPSO-LR results to be the most precise, respectively.

Analyzing the algorithms for the multimodal benchmark functions $(f_9-\ f_{15})$, there is considerable variation in accuracy and precision. However, by considering these features separately, the most accurate, but not necessarily the most precise algorithms in descendant order are: QPSO-LR, GA, PSO, FFO, QPSO-CS, QPSO-RM. In contrast, the algorithms that are more precise, but not necessarily the most accurate in descendant order are: QPSO-RM, FFO, GA, QPSO-LR, QPSO-CS, and PSO.

**Table 1:** Accuracy and precision metrics for unimodal benchmark functions with N = 30, Tmax = 1000 and Texp = 30.

Function	Metrics	QPSO-LR	QPSO-RM	QPSO-CS	PSO	FFO	GA
$f_{1}$	$\delta$	$4.0379 \times 10^{- 27}$	0.0011	$3.1073 \times 10^{- 32}$	$5.9653 \times 10^{- 19}$	$4.8991 \times 10^{- 8}$	0.1105
	$\phi$	1.5526	2.4002	2.0483	1.6922	0.1274	0.6321
$f_{2}$	$\delta$	$4.1492 \times 10^{- 19}$	0.5925	$2.1296 \times 10^{- 7}$	$2.8087 \times 10^{- 6}$	21.6909	0.0685
	$\phi$	0.9150	1.1420	2.7324	5.4564	1.6504	0.5758
$f_{3}$	$\delta$	87.8039	$5.4345 \times 10^{3}$	123.5104	8.7609	$1.2248 \times 10^{3}$	$2.9055 \times 10^{3}$
	$\phi$	0.4933	0.3378	0.5219	0.7939	0.4788	0.4615
$f_{4}$	$\delta$	0.0126	31.6220	0.5792	0.4655	26.0576	4.7834
	$\phi$	0.5341	0.1802	0.3986	0.4387	0.2965	0.2278
$f_{5}$	$\delta$	66.0430	668.2517	43.0373	51.0794	188.8109	142.7954
	$\phi$	0.9455	0.5255	1.0665	0.8570	1.5627	0.6277
$f_{6}$	$\delta$	0	866.6000	3.8667	0.2667	9.4000	0.0333
	$\phi$	0	0.6728	3.2612	2.9434	0.8686	5.4772
$f_{7}$	$\delta$	0.0170	0.4136	0.0198	0.0153	0.9466	0.0253
	$\phi$	0.4261	0.6762	0.3989	0.6051	0.5203	0.3168
$f_{8}$	$\delta$	5.5352	6.3865	4.9975	2.8966	11.3206	1.5176
	$\phi$	0.2822	0.2962	0.3784	0.6235	0.1053	0.3179

**Table 2:** Accuracy and precision metrics for multimodal benchmark functions with N = 30, Tmax = 1000 and Texp = 30.

Function	Metrics	QPSO-LR	QPSO-RM	QPSO-CS	PSO	FFO	GA
$f_{9}$	$\delta$	$1.0377 \times 10^{4}$	$7.5089 \times 10^{3}$	$9.4712 \times 10^{3}$	$2.4246 \times 10^{4}$	$5.4177 \times 10^{3}$	$1.2561 \times 10^{4}$
	$\phi$	0.0449	0.0747	0.0598	0.1706	$3.4152 \times 10^{- 16}$	$4.6904 \times 10^{- 4}$
$f_{10}$	$\delta$	24.1505	82.0155	61.3556	54.1920	193.2863	0.6408
	$\phi$	0.3292	0.2662	0.2714	0.2700	0.1876	1.0218
$f_{11}$	$\delta$	$4.0679 \times 10^{- 14}$	8.6475	1.7671	0.8314	19.9668	0.1446
	$\phi$	0.5166	0.1931	0.5818	0.9961	$5.5842 \times 10^{- 11}$	0.5556
$f_{12}$	$\delta$	0.0172	1.2919	0.0330	0.0136	0.0087	0.2078
	$\phi$	1.2614	1.1735	1.3155	1.2714	1.3300	0.4605
$f_{13}$	$\delta$	0.0173	14.5092	0.1630	0.1349	16.5333	0.0016
	$\phi$	2.2743	0.5130	2.0342	1.9827	0.4402	3.6775
$f_{14}$	$\delta$	0.0015	33.5424	0.0288	0.0569	13.9971	0.0082
	$\phi$	2.5931	0.3051	4.2382	5.1166	1.3473	1.1503
$f_{15}$	$\delta$	$1.1676 \times 10^{- 13}$	1.7340	$3.4012 \times 10^{- 8}$	$7.2088 \times 10^{- 9}$	2.8835	0.0047
	$\phi$	1.0440	0.9820	4.9304	2.8034	0.5131	0.3573

**Table 3:** Accuracy and precision metrics for fixed-multimodal benchmark functions with Tmax = 1000 and Texp = 30.

Function	Metrics	QPSO-LR	QPSO-RM	QPSO-CS	PSO	FFO	GA
$f_{16}$	$\delta$	0.6460	0.6624	0.5710	0.5531	0.4058	0.4688
	$\phi$	0.2617	0.2410	0.2592	0.2439	0.3711	0.4651
$f_{17}$	$\delta$	0.0023	0.0011	0.0023	$2.2142 \times 10^{- 4}$	0.0060	0.0067
	$\phi$	2.3522	2.5454	2.3449	0.6455	1.9599	1.3437
$f_{18}$	$\delta$	$1.5465 \times 10^{- 6}$	$1.5465 \times 10^{- 6}$	$1.5465 \times 10^{- 6}$	$1.5465 \times 10^{- 6}$	$1.5465 \times 10^{- 6}$	$1.3332 \times 10^{- 4}$
	$\phi$	$6.4438 \times 10^{- 16}$	$6.3831 \times 10^{- 16}$	$6.5062 \times 10^{- 16}$	$6.5675 \times 10^{- 16}$	$1.813 \times 10^{- 15}$	$4.0007 \times 10^{- 4}$
$f_{19}$	$\delta$	$1.1264 \times 10^{- 4}$	$1.1264 \times 10^{- 4}$	$1.1264 \times 10^{- 4}$	$1.1264 \times 10^{- 4}$	0.0768	0.0011
	$\phi$	0	0	0	0	0.8873	0.0066
$f_{20}$	$\delta$	$7.9491 \times 10^{- 14}$	$7.7272 \times 10^{- 14}$	$7.9491 \times 10^{- 14}$	$7.8601 \times 10^{- 14}$	$1.5986 \times 10^{- 14}$	0.0083
	$\phi$	$4.6649 \times 10^{- 16}$	$3.7488 \times 10^{- 16}$	$5.2010 \times 10^{- 16}$	$1.8436 \times 10^{- 16}$	$8.7753 \times 10^{- 15}$	0.0087
$f_{21}$	$\delta$	$1.7852 \times 10^{- 5}$	$1.7852 \times 10^{- 5}$	$1.7852 \times 10^{- 5}$	$1.7852 \times 10^{- 5}$	$1.7852 \times 10^{- 5}$	$5.0918 \times 10^{- 5}$
	$\phi$	$7.0163 \times 10^{- 16}$	$6.9801 \times 10^{- 16}$	$7.0159 \times 10^{- 16}$	$7.0159 \times 10^{- 16}$	$3.9481 \times 10^{- 16}$	$1.8331 \times 10^{- 5}$
$f_{22}$	$\delta$	0.0495	0.0456	0.0535	0.0535	0.1030	0.0496
	$\phi$	0.0183	0.0181	0.0185	0.0185	0.0131	0.0183
$f_{23}$	$\delta$	3.3818	4.2363	4.2274	2.6751	4.7546	3.2649
	$\phi$	0.4677	0.5803	0.5433	0.4643	0.5808	0.4994
$f_{24}$	$\delta$	4.5851	3.8040	3.5104	1.3978	4.7708	2.2613
	$\phi$	0.6081	0.5526	0.5492	0.3164	0.6589	0.4241

For fixed multimodal functions $(f_{16}-f_{24})$, the results expose that QPSO-LR and QPSO-RM show excellent results for functions $f_{16}$ and $f_{19}$ in terms of accuracy and precision, while PSO responds better to $f_{17}$. For the rest of the functions, all algorithms present acceptable accuracy and precision.

### Exploration: speed and acceleration

**Figure 6:**
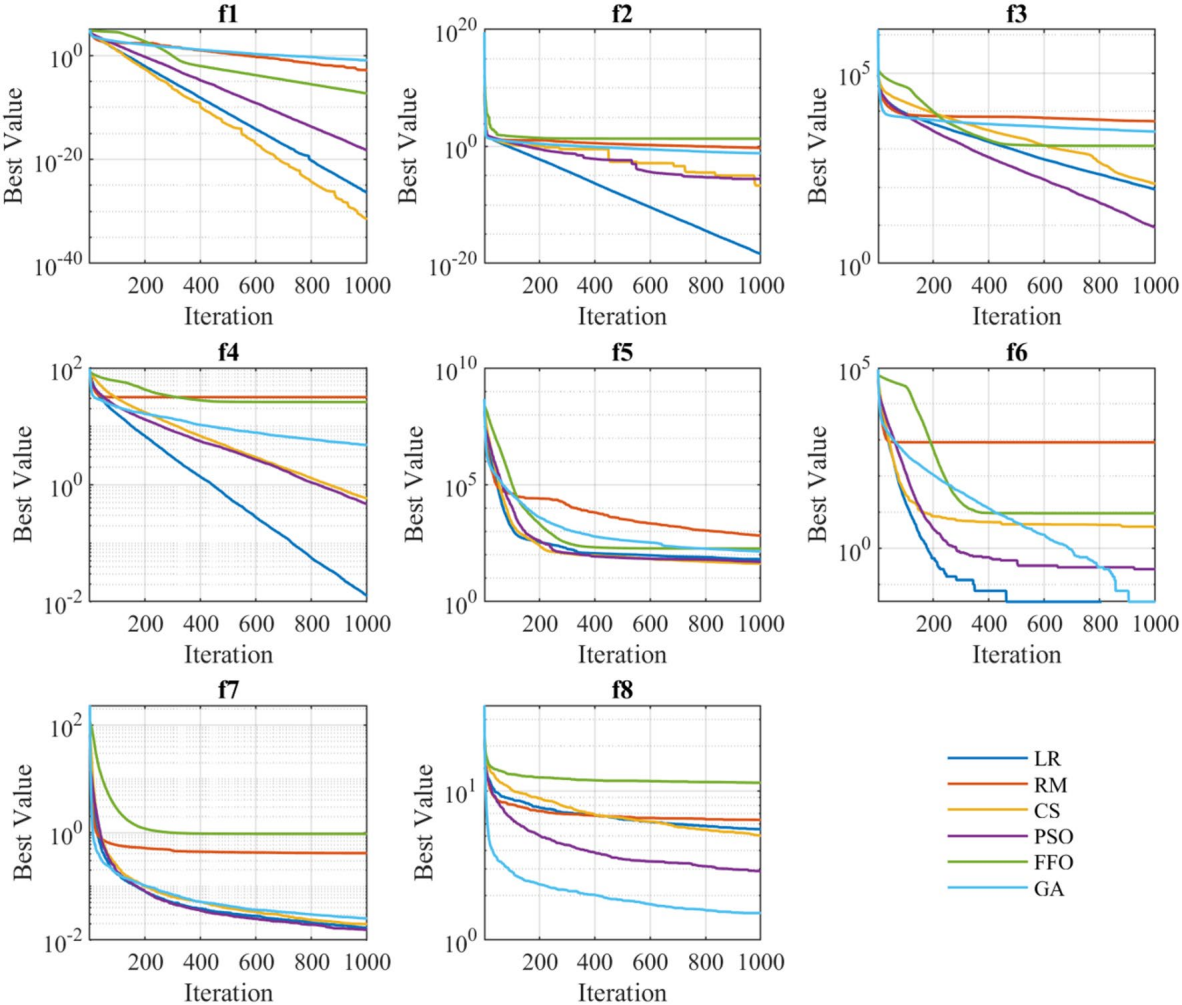
Convergence behaviour of the unimodal benchmark functions.

**Figure 7:**
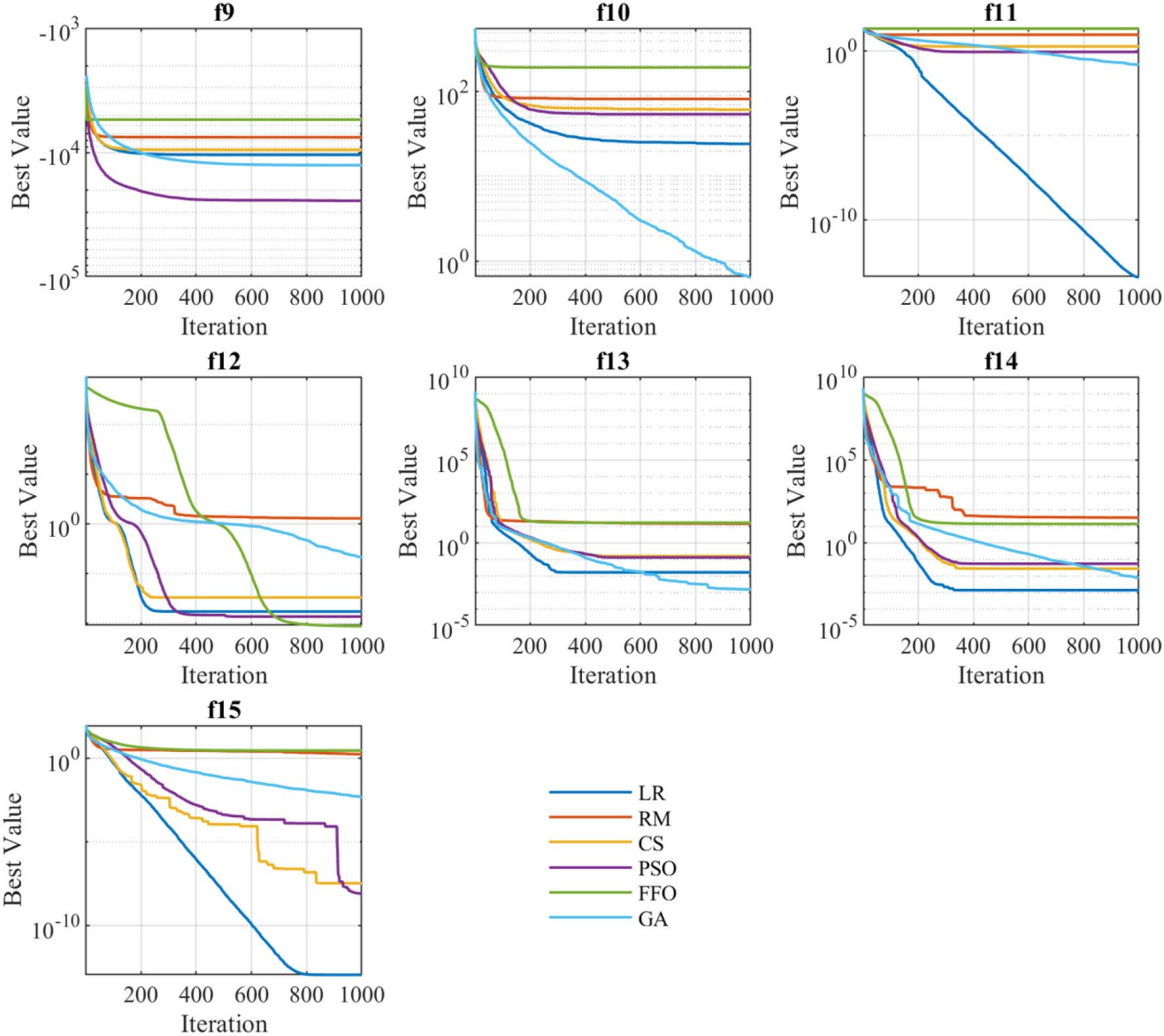
Convergence behaviour of the multimodal benchmark functions.

**Figure 8:**
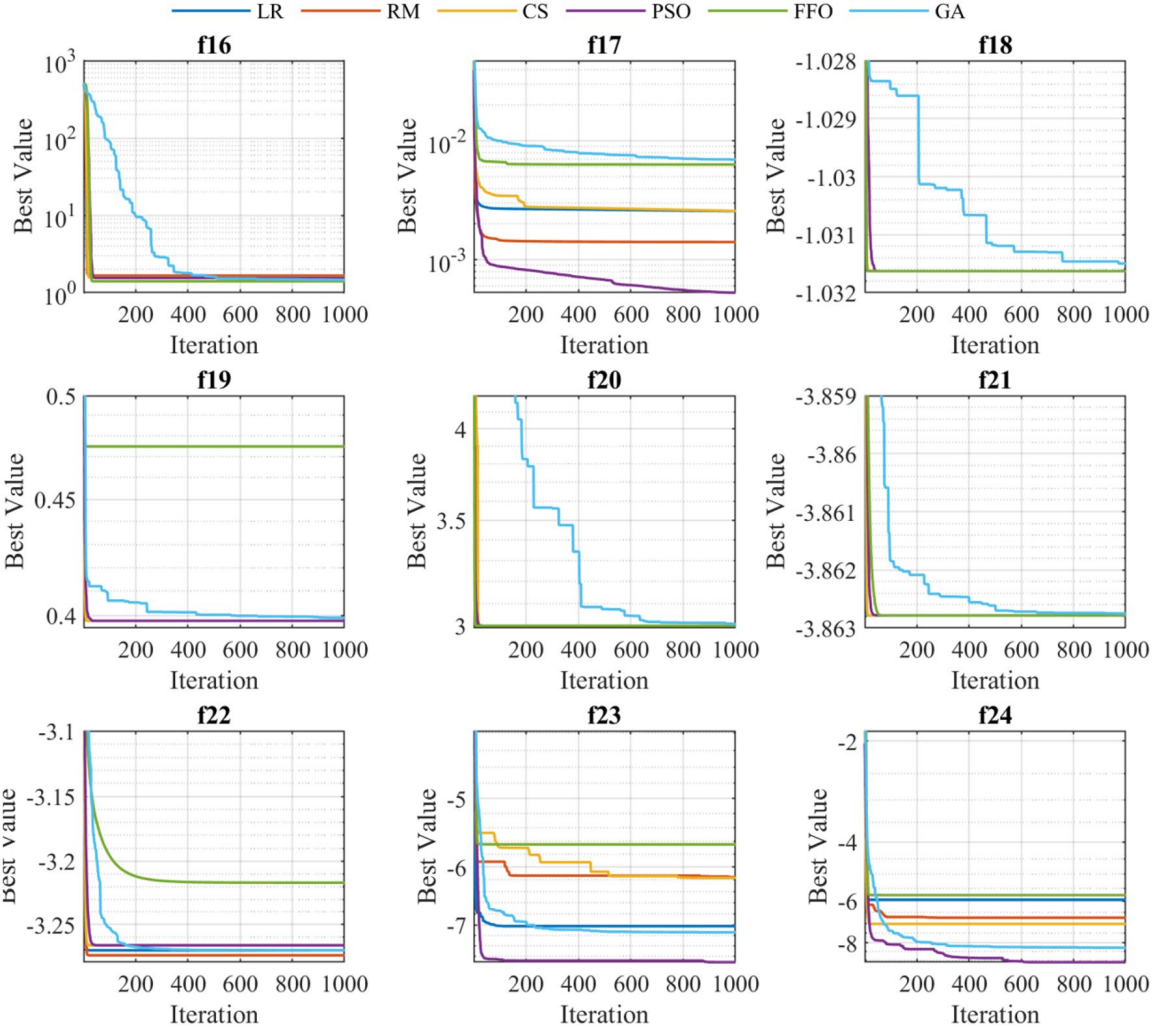
Convergence behaviour of the fixed-multimodal benchmark functions.

The exploration is defined as the ability of examining the promising area(s) of the search space as broadly as possible^56^. The exploration is closed related with the convergence behaviour, which is shown in Figs. 6, 7 and 8. These graphs represent the evolution of the best solution through every iteration performed. It can be appreciated that depending on the type of function on which the algorithms are applied, presents certain patterns. For instance, functions $f_1,\ f_2,\ f_3,\ f_4,\ f_5$ (independently of the employed algorithm) present a linear convergence behaviour, while for the rest of functions the behaviour is exponential.

To quantify the exploration, the average search speed and acceleration of each algorithm is calculated using the Allan variances^58^. The first Allan variance index measures search speed, i.e. the distance variation of best search agent C in every iteration k, which is mathematically described in Eq. (29). The second Allan variation index measures the search acceleration, i.e. the search speed variation, which is mathematically described in Eq. (30).

29 $$\begin{aligned} \omega= & {} \left| \frac{\sum _{k=1}^{k_{max}}{C\left( k+1\right) -C\left( k\right) }}{\Delta k}\right| \end{aligned},$$

30 $$\begin{aligned} \alpha= & {} \left| \frac{\sum _{k=1}^{k_{max}}{\omega \left( k+1\right) -\omega \left( k\right) }}{\Delta k}\right| \end{aligned}.$$

Tables 4, 5 and 6 shows the search speed and acceleration for each optimization technique grouped by unimodal, multimodal, and fixed multimodal functions, respectively (best values are highlighted in blue). For all types of functions, GA algorithm exhibits the highest degree of average search speed and acceleration. However, speed and acceleration attributes does not assure good precision, accuracy, or simulation time. Therefore, a more scrutiny analysis is required to develop a proper comparison between optimization methods, as presented in the next sections.

**Table 4:** Convergence speed and acceleration metrics for unimodal benchmark functions with N = 30, Tmax = 1000 and Texp = 30.

Function	Metrics	QPSO-LR	QPSO-RM	QPSO-CS	PSO	FFO	GA
$f_{1}$	$\omega$	$1.4443 \times 10^{3}$	$1.5990 \times 10^{3}$	$1.7382 \times 10^{3}$	$5.9047 \times 10^{2}$	$4.7911 \times 10^{2}$	$3.3144 \times 10^{3}$
	$\alpha$	$3.4075 \times 10^{2}$	$4.2430 \times 10^{2}$	$3.3948 \times 10^{2}$	6.0829	5.2972	$2.0046 \times 10^{3}$
$f_{2}$	$\omega$	$4.1564 \times 10^{7}$	$7.1210 \times 10^{7}$	$3.8718 \times 10^{2}$	$5.1176 \times 10^{2}$	$5.1176 \times 10^{10}$	$1.1566 \times 10^{18}$
	$\alpha$	$4.2608 \times 10^{7}$	$7.2078 \times 10^{7}$	$7.6250 \times 10^{8}$	$6.3001 \times 10^{- 2}$	$3.6744 \times 10^{10}$	$1.1979 \times 10^{18}$
$f_{3}$	$\omega$	$2.2971 \times 10^{3}$	$2.6881 \times 10^{3}$	$2.3607 \times 10^{3}$	$9.8210 \times 10^{2}$	$1.7612 \times 10^{3}$	$4.7806 \times 10^{4}$
	$\alpha$	$3.3765 \times 10^{2}$	$5.8023 \times 10^{2}$	$5.2257 \times 10^{2}$	51.6621	$1.4315 \times 10^{2}$	$4.6678 \times 10^{4}$
$f_{4}$	$\omega$	1.5963	1.5752	1.0108	0.7047	0.4801	2.3429
	$\alpha$	0.1307	0.1186	0.0329	0.0237	0.0132	0.8982
$f_{5}$	$\omega$	$4.4969 \times 10^{6}$	$4.7306 \times 10^{6}$	$6.3133 \times 10^{6}$	$1.0640 \times 10^{6}$	$7.5646 \times 10^{6}$	$1.6173 \times 10^{7}$
	$\alpha$	$1.9421 \times 10^{6}$	$1.6195 \times 10^{6}$	$1.4387 \times 10^{6}$	$6.5665 \times 10^{2}$	$2.0199 \times 10^{5}$	$1.3257 \times 10^{7}$
$f_{6}$	$\omega$	$1.5644 \times 10^{3}$	$1.5276 \times 10^{3}$	$1.7360 \times 10^{3}$	$5.9708 \times 10^{2}$	$4.7453 \times 10^{2}$	$3.0891 \times 10^{3}$
	$\alpha$	$4.8047 \times 10^{2}$	$3.8717 \times 10^{2}$	$2.9077 \times 10^{2}$	9.3619	9.2238	$1.8074 \times 10^{3}$
$f_{7}$	$\omega$	2.1368	2.3284	3.0930	0.4842	3.2862	7.9698
	$\alpha$	0.7444	0.8915	0.6685	0.0040	0.0829	6.5391
$f_{8}$	$\omega$	0.34833	0.4149	0.3111	0.1649	0.3147	1.1468
	$\alpha$	0.0707	0.0842	0.0743	0.0041	0.0278	0.6206

**Table 5:** Accuracy and precision metrics for multimodal benchmark functions with N = 30, Tmax = 1000 and Texp = 30.

Function	Metrics	QPSO-LR	QPSO-RM	QPSO-CS	PSO	FFO	GA
$f_{9}$	$\omega$	$1.4137 \times 10^{2}$	$1.4873 \times 10^{2}$	$1.3877 \times 10^{2}$	$2.6002 \times 10^{2}$	90.5291	$1.8003 \times 10^{2}$
	$\alpha$	6.0517	6.7619	0.8768	28.8637	80.4336	90.3119
$f_{10}$	$\omega$	7.1803	9.1616	6.6542	2.4200	7.2001	14.7343
	$\alpha$	0.6570	1.1580	0.7452	0.0361	0.4907	6.6963
$f_{11}$	$\omega$	0.3426	0.3647	0.3127	0.2036	0.0001	0.0516
	$\alpha$	0.0116	0.0167	$1.688 \times 10^{- 4}$	0.0045	0.0001	19.2122
$f_{12}$	$\omega$	12.9950	14.1370	15.6384	5.2224	4.3928	30.8202
	$\alpha$	2.9750	3.7139	2.5028	0.0400	0.0820	19.2122
$f_{13}$	$\omega$	$1.0457 \times 10^{7}$	$8.9922 \times 10^{6}$	$1.3693 \times 10^{7}$	$1.2975 \times 10^{6}$	$9.9214 \times 10^{6}$	$4.5856 \times 10^{7}$
	$\alpha$	$4.6454 \times 10^{6}$	$4.2749 \times 10^{6}$	$3.1766 \times 10^{6}$	$2.7464 \times 10^{3}$	$2.8049 \times 10^{5}$	$4.1327 \times 10^{7}$
$f_{14}$	$\omega$	$1.9438 \times 10^{7}$	$1.9614 \times 10^{7}$	$2.7256 \times 10^{7}$	$3.4698 \times 10^{6}$	$1.6631 \times 10^{7}$	$7.3698 \times 10^{7}$
	$\alpha$	$7.4400 \times 10^{6}$	$8.2036 \times 10^{6}$	$7.1400 \times 10^{6}$	$1.3277 \times 10^{4}$	$5.1217 \times 10^{5}$	$6.1365 \times 10^{7}$
$f_{15}$	$\omega$	1.4280	1.6201	1.3598	0.5925	1.3838	2.7137
	$\alpha$	0.2589	0.1861	0.0947	0.0103	0.0773	1.2823

**Table 6:** Accuracy and precision metrics for fixed-multimodal benchmark functions with N = 30, Tmax = 1000 and Texp = 30.

Function	Metrics	QPSO-LR	QPSO-RM	QPSO-CS	PSO	FFO	GA
$f_{16}$	$\omega$	17.1710	17.1800	17.1860	17.1130	17.1860	6.2410
	$\alpha$	0.5586	0.0081	0.0088	0.0802	0.0080	0.2768
$f_{17}$	$\omega$	0.0014	0.0019	0.0020	0.0013	0.0030	$1.8499 \times 10^{2}$
	$\alpha$	0.0008	0.0012	0.0012	0.0007	0.0011	$1.9159 \times 10^{2}$
$f_{18}$	$\omega$	0.0090	0.0128	0.0155	0.0005	0.0151	32.3928
	$\alpha$	0.0062	0.0086	0.0109	0.0019	0.0070	33.5288
$f_{19}$	$\omega$	0.0186	0.0241	0.0200	0.0114	0.0306	1.9380
	$\alpha$	0.0107	0.0173	0.0130	0.0042	0.0007	1.9688
$f_{20}$	$\omega$	0.3086	0.5306	0.4182	0.3813	0.2147	$3.1195 \times 10^{3}$
	$\alpha$	0.1404	0.3354	0.2157	0.2384	0.1398	$3.2301 \times 10^{3}$
$f_{21}$	$\omega$	0.0040	0.0067	0.0057	0.0036	0.0032	0.1029
	$\alpha$	0.0025	0.0041	0.0032	0.0012	0.0022	0.0998
$f_{22}$	$\omega$	0.0351	0.0338	0.0323	0.0215	0.0217	0.1005
	$\alpha$	0.0168	0.0112	0.0132	0.0064	0.0064	0.0702
$f_{23}$	$\omega$	0.1875	0.1610	0.1512	0.1948	0.1671	0.1919
	$\alpha$	0.0469	0.0178	0.0193	0.0058	0.0018	0.0337
$f_{24}$	$\omega$	0.1552	0.1682	0.1937	0.1922	0.1640	0.1606
	$\alpha$	0.0174	0.0156	0.0200	0.0060	0.0025	0.0329

### Simulation time

**Figure 9:**
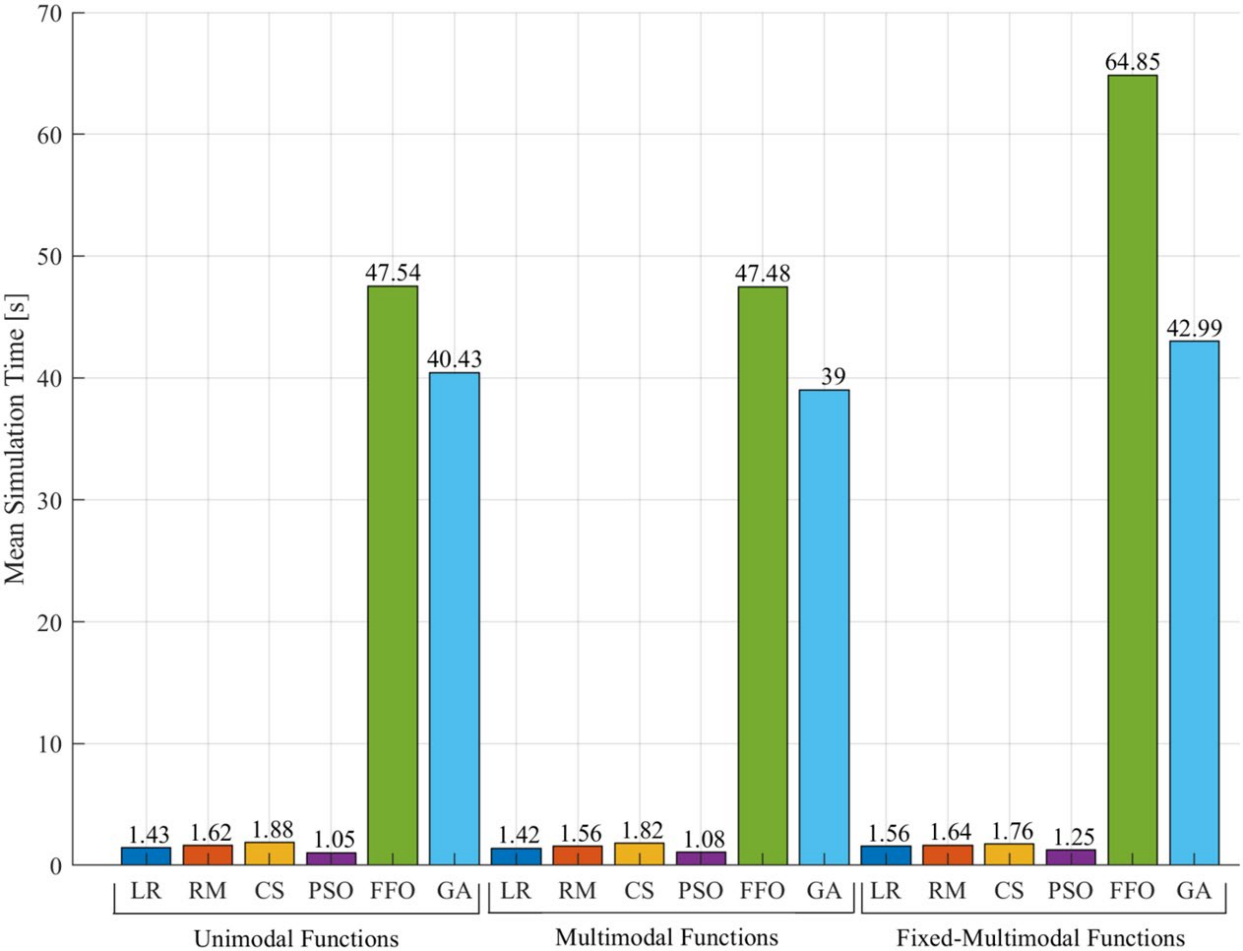
Average simulation time.

Another important aspect to evaluate is the simulation time performance. Figure  9 shows the average execution time for each optimization algorithm. The results reveal that FFO and GA present higher time simulation (exceeds in approximately between 34 to 48 times) compared with the rest of the optimization techniques. Therefore, QPSO-LR, QPSO-RM, QPSO-CS and PSO present better performance in terms of simulation time than FFO and GA.

### Overall performance

The performance in terms of accuracy, precision, search speed, search acceleration, and simulation time of each optimization technique is quantitative defined by the grade rules presented in (31) to (35), respectively. These rules are developed to facilitate the comparison between optimization techniques under the following criterion: ‘+ 3’ excellent performance, ‘+ 2’ good performance, ‘+ 1’ fair performance, ‘+ 0’ low performance. Once the values are assigned, the average of the function by groups (unimodal, multimodal, fixed multimodal) is taken. Then, the values are normalized based on the highest average.

31 $$rule_{\delta } = \left\{ {\begin{array}{*{20}l} { + 3} \hfill & {if\;\delta < 1 \times 10^{{ - 6}} } \hfill \\ { + 2} \hfill & {if\;1 \times 10^{{ - 6}} \le \delta < 1 \times 10^{{ - 3}} } \hfill \\ { + 1} \hfill & {if\;1 \times 10^{{ - 3}} \le \delta < 1} \hfill \\ 0 \hfill & {if\;\delta \ge 1} \hfill \\ \end{array},} \right.$$

32 $$rule_{\sigma } = \left\{ {\begin{array}{*{20}l} { + 3} \hfill & {if\;\sigma < 1 \times 10^{{ - 3}} } \hfill \\ { + 2} \hfill & {if\;1 \times 10^{{ - 3}} \le \sigma < 1} \hfill \\ { + 1} \hfill & {if\;1 \le \sigma < 3} \hfill \\ 0 \hfill & {if\;\delta \ge 3} \hfill \\ \end{array},} \right.$$

33 $$rule_{\omega } = \left\{ {1\begin{array}{*{20}l} { + 3} \hfill & {if\;\omega > 1 \times 10^{6} } \hfill \\ { + 2} \hfill & {if\;1 \times 10^{3} < \omega \le 1 \times 10^{6} } \hfill \\ { + 1} \hfill & {if\;1 < \omega \le 1 \times 10^{3} } \hfill \\ 0 \hfill & {if\;\omega \le 3} \hfill \\ \end{array},} \right.$$

34 $$rule_{\alpha } = \left\{ {1\begin{array}{*{20}l} { + 3} \hfill & {if\;\alpha > 1000} \hfill \\ { + 2} \hfill & {if\;100 < \alpha \le 1000} \hfill \\ { + 1} \hfill & {if\;1 < \alpha \le 100} \hfill \\ 0 \hfill & {if\;\alpha \le 1} \hfill \\ \end{array},} \right.$$

35 $$rule_{\tau } = \left\{ {\begin{array}{*{20}l} { + 3} \hfill & {if\;\tau < 2} \hfill \\ { + 2} \hfill & {if\;2 \le \tau < 4} \hfill \\ { + 1} \hfill & {if\;4 \le \tau < 6} \hfill \\ 0 \hfill & {if\;\tau \ge 6} \hfill \\ \end{array},} \right.$$

**Figure 10:**
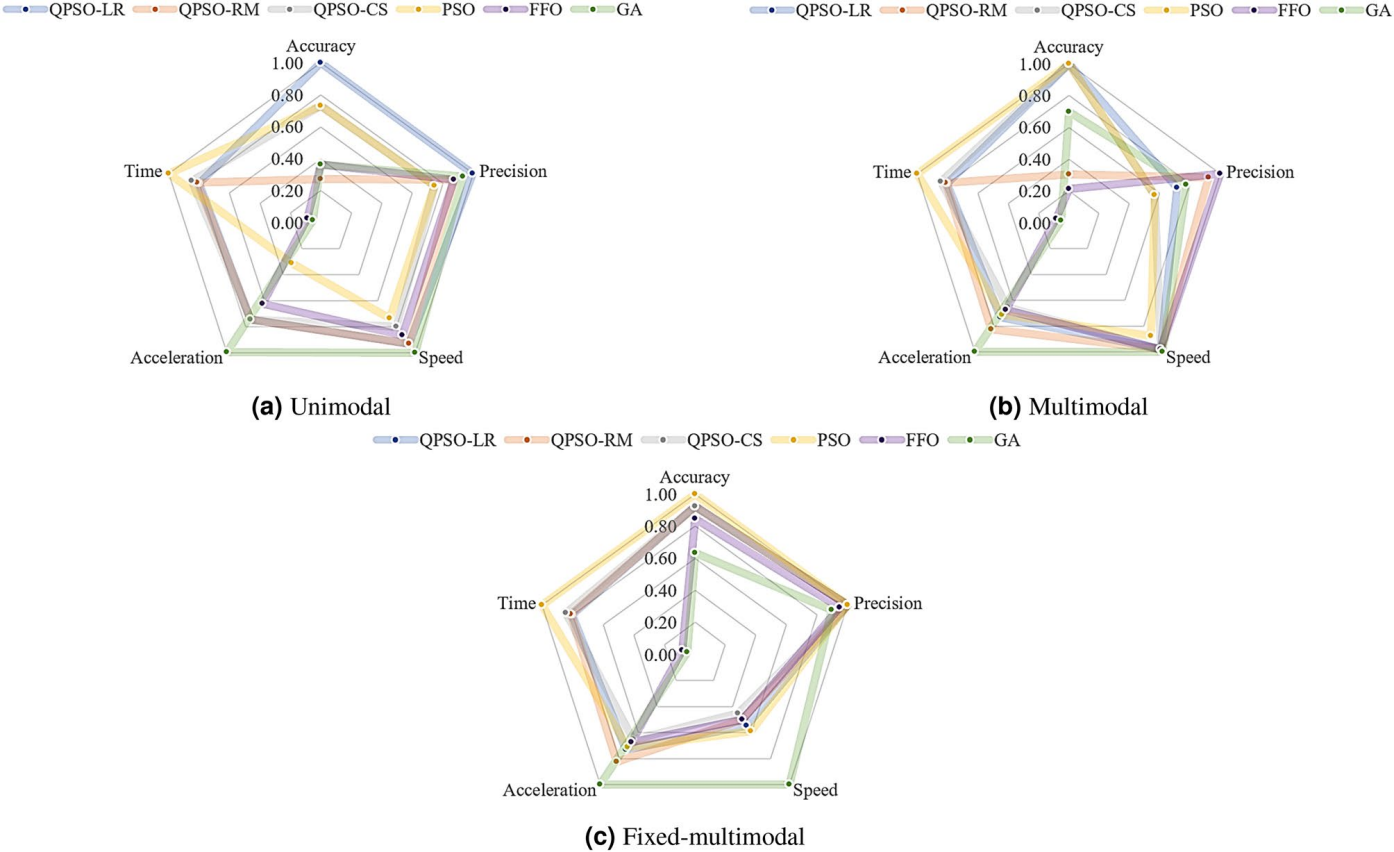
Optimization techniques performance categorized by type of function.

Finally, the results are integrated into a spider-chart as shown in Fig. 10 to show the overall performance of each optimization technique.

As a general trend, it can be seen in Fig. 10 that for the three types of functions the algorithm that is closest on average to 100% performance in all five indexes is the QPSO- LR, followed by the PSO, the QPSO- CS and finally the GA and FFA method. A closer examination reveals:

- With respect to accuracy, the QPSO-LR, PSO and QPSO-CS go first, second and third respectively with an average performance of 97%, 91% and 88%.
- In terms of precision the FFO, the QPSO-LR and GA can be ranked as first, second and third, respectively with an average performance over the three type of functions of 94%, 91%, and 87%.
- Referring to speed of convergence, GA technique can be ranked first with 100% average performance, followed by the QPSO-LR, 83% and the QPSO-RM, 80%.
- In terms of acceleration of convergence GA has 100%, followed by QPSO-RM and QPSO-LR with 80% and 74% of average performance, respectively.
- Regarding the simulation time, PSO can be ranked at the top with an average performance of 100% being followed by the QPSO-CS, QPSO-RM and the QPSO-LR with an average performance between 85% and 80%.

To get a better understanding of the performance in terms of exploitation, the accuracy and precision are averaged for each type of function (Unimodal, Multimodal and Fixed multimodal). The same process is done with search speed and acceleration to quantify exploration (see Table 10). As a result, the exploitation performance of QPSO-LR is ranked first, followed by the QPSO-RM and the QPSO-CS (just considering the proposed approaches). This is expected since the Lorentz potential field is the weakest as $(z \rightarrow \pm \infty )$ and the Coulomb like potential diverges as $(z \rightarrow \pm \infty )$, being the strongest. Regarding exploration the QPSO-RM is ranked first, followed by the QPSO-LR and the QPSO-CS. Again, this is also expected since the Rosen Morse potential field is the strongest in between the limits of the quantum well and the Coulomb like potential diverges towards $-\infty$, being the weakest (steepest too).

An increase in exploration and a slightly decrease in exploitation is observed in the multimodal functions compared to the unimodal, due to the high number of traps that may exist in the hypersurface being searched. Also, an increase in exploitation and decrease in exploration are observed in the fixed multimodal functions compared to unimodal, attributed to irregular hypersurface formed. While the behaviour of the search algorithm may change for each type of function, the QPSO-LR always exhibited higher exploitation and moderate-high exploration. Being the combination of weak potential at $(z \rightarrow \pm \infty )$ and moderate steepness between the limits of the quantum well that are responsible of such trend.

## Discussion and conclusion

Three novel quantum-behaved swarm optimization algorithms based on Lorentz (QPSO-LR), Rosen–Morse (QPSO-RM) and Coulomb-like Square Root (QPSO-CS) potential fields are proposed. The QPSO-LR, QPSO-RM, and QPSO-CS are inspired in the models of donor acceptor interaction between particles, molecular vibrations, and electron confinement in graphene, respectively.

To verify the efficacy of the proposed optimization techniques, twenty-four test functions grouped by unimodal, multimodal, fixed multimodal are employed to benchmark their performance in terms of exploration (accuracy and precision), exploitation (search speed and acceleration), and simulation time. The results show that the proposed approaches (QPSO-LR, QPSO-RM, and QPSO-CS) present several advantages in comparison to traditional optimization techniques, such as, PSO, FFO, and GA. For instance, QPSO-LR exhibits better accuracy performance than PSO, FFA, and GA, for all the type functions; QPSO-CS shows better precision than GA and PSO in overall; QPSO-RM and QPSO-LR have a faster speed and acceleration of search than PSO and FFO; QPSO-LR, QPSO-RM, and QPSO-CS show significant computation time performance than FFO and GA.

Among the proposed optimizations techniques, there is not one that show the best performance in all the given attributes (exploration, exploitation, and simulation time), however, QPSO-LR is the most balanced, which makes it a powerful global searcher for different applications. Therefore, the aim of future research is to investigate the use of QPSO-LR combined with other computational intelligence methodologies, such as fuzzy systems and neural networks for optimization in multivariable processes in order to enhance the performance of the approach.

## Figures and Tables

**Table 7:**
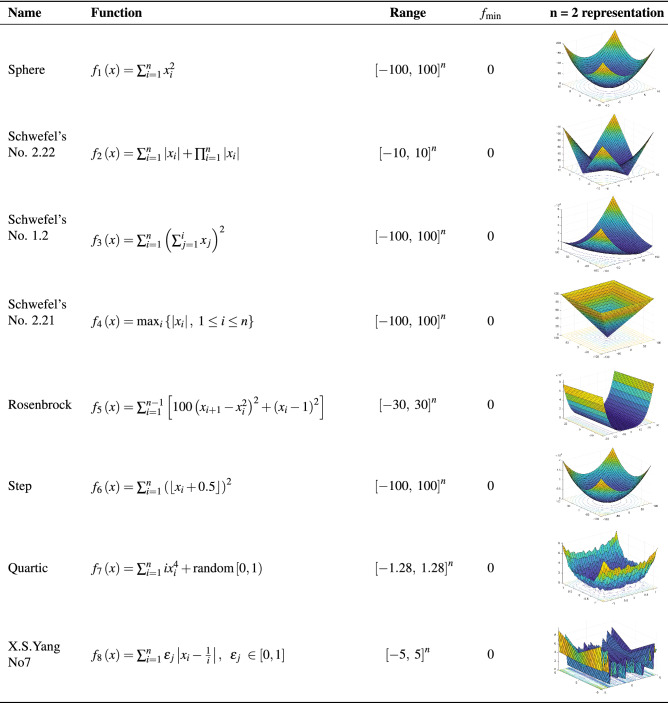
Unimodal benchmark functions.

**Table 8:**
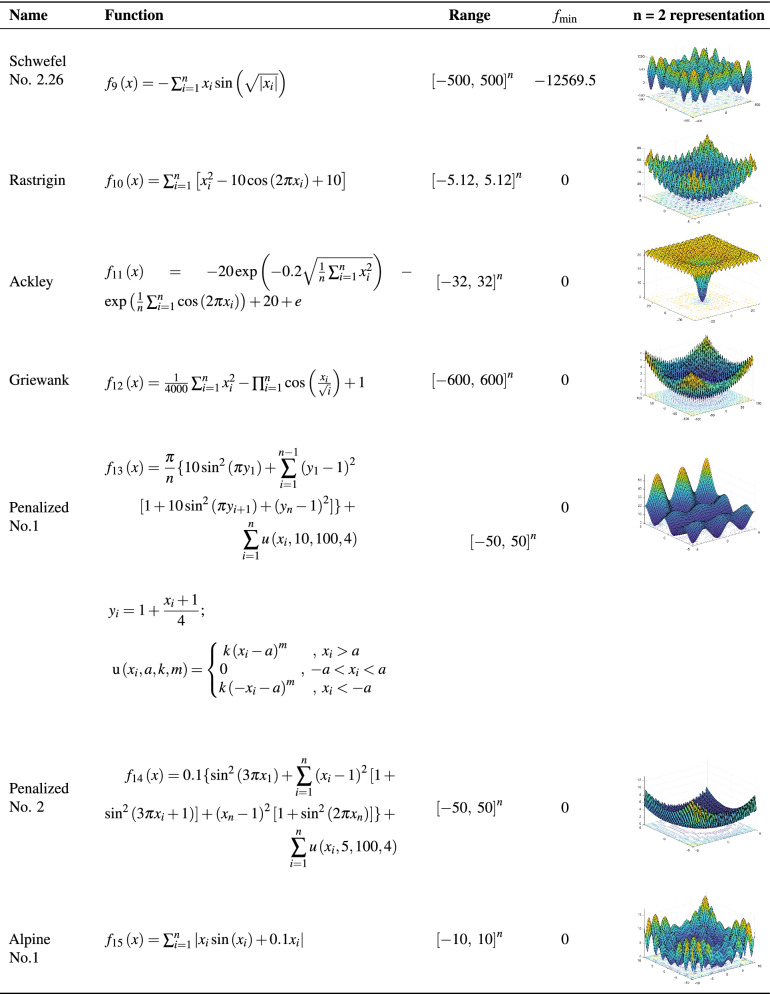
Multimodal benchmark functions.

**Table 9:**
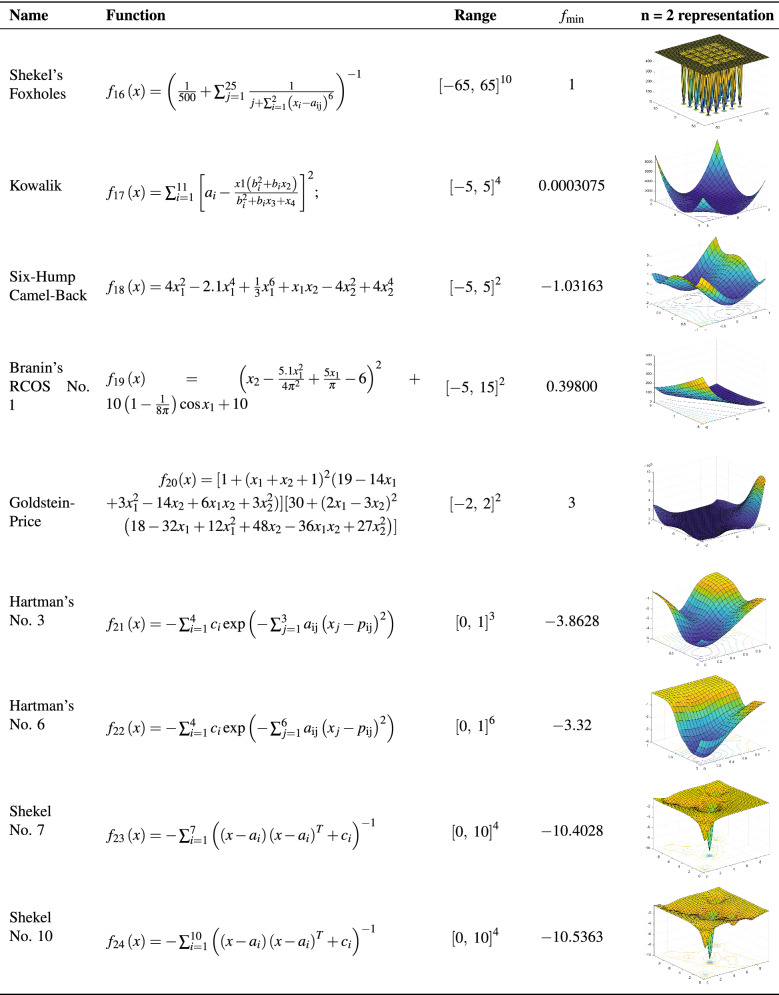
Fixed-dimension multimodal benchmark functions.

## Case study: benchmark functions

See Tables 7, 8 and 9.

## Overall performance of exploitation and exploration of the proposed approaches

See Table 10.

**Table 10:** Exploitation and exploration performance.

Function type	Search algorithm	Exploitation	Exploration
Unimodal	QPSO-LR	1.00	0.84
	QPSO-CS	0.60	0.74
	QPSO-RM	0.60	0.84
Multimodal	QPSO-LR	0.86	0.86
	QPSO-CS	0.60	0.83
	QPSO-RM	0.62	0.90
Fixed multimodal	QPSO-LR	0.96	0.64
	QPSO-CS	0.90	0.60
	QPSO-RM	0.96	0.66
